# Hungarian Validation of the Pelvic Floor Distress Inventory - 20 (PFDI-20) Questionnaire and the Pelvic Floor Impact Questionnaire - 7 (PFIQ-7)

**DOI:** 10.1007/s00192-025-06437-y

**Published:** 2025-11-15

**Authors:** Barbara Kozma, Bence Kozma, Peter Takacs, Dávid Rátonyi, Nelli Farkas, Attila G. Sipos, Bálint Farkas

**Affiliations:** 1 Faculty of Medicine, Department of Obstetrics and Gynecology, University of Debrecen, Nagyerdei krt. 98., 4032 Debrecen, Hungary; 2 Doctoral School of Nutrition and Food Sciences, University of Debrecen, University of Egyetem, tér 1., 4032 Debrecen, Hungary; 3 Eastern Virginia Medical School, Department of Obstetrics and Gynecology, Division of Female Pelvic Medicine and Reconstructive Surgery, 825 Fairfax Avenue, Suite 526, Norfolk, Virginia 23507, 2007 USA; 4 Faculty of Medicine Institute of Bioanalysis, University of Pécs, Honvég str. 1., 7624 Pécs, Hungary; 5 Faculty of Medicine, Department of Obstetrics and Gynecology, University of Pécs, Édesanyák str. 17., 7624 Pécs, Hungary; 6 National Laboratory on Human Reproduction, University of Pécs, Pécs, Hungary

**Keywords:** Pelvic Floor Distress Inventory Questionnaire – Short Form 20 (PFDI-20) 1, Pelvic Floor Disorder (PFD) 2, Pelvic Floor Impact Questionnaire - Short Form 7 (PFIQ-7) 3, Quality of life 4, Questionnaire validation 5

## Abstract

**Introduction and Hypotheses:**

Pelvic floor disorders (PFDs) affect women’s quality of life through physical, psychological, and social domains. The Pelvic Floor Distress Inventory-20 (PFDI-20) and the Pelvic Floor Impact Questionnaire-7 (PFIQ-7) are validated tools for assessing symptom severity and quality of life impact. The current study aim to investigate the reliability and validity of the Hungarian versions of these questionnaires in women with PFD symptoms.

**Methods:**

This validation study was carried out at the University of Debrecen among women with clinically confirmed pelvic floor disorders. The PFDI-20 and PFIQ-7 questionnaires were translated into Hungarian using independent native English speaker in the back- translation process. Psychometric testing included internal consistency (Cronbach’s alpha), test–retest reliability (ICC), and construct validity using the SF-36 Health Survey. The tools were valid and reliable for assessing pelvic floor symptoms and their impact on the quality of life in Hungarian women.

**Results:**

One hundred fifty patients completed the Hungarian PFDI-20, PFIQ-7, and SF-36 questionnaires for psychometric evaluation. Both Hungarian versions showed excellent internal consistency (PFDI-20 *α* = 0.885; PFIQ-7 *α* = 0.939) and strong test–retest reliability (PFDI-20 ICC = 0.903; PFIQ-7 ICC = 0.933).

**Discussion:**

The Hungarian versions of the PFDI-20 and PFIQ-7 questionnaires demonstrated excellent reliability, validity, and cultural relevance for assessing symptom severity and quality of life in women with pelvic floor disorders. Strong internal consistency, high test–retest reliability, and significant correlations with the SF-36 support their psychometric soundness. These tools are suitable for both clinical and research applications in Hungarian-speaking populations.

## Introduction

Female pelvic floor disorders encompass a range of clinical conditions, including urinary and fecal incontinence, pelvic organ prolapse, sensory and emptying dysfunctions of the lower urinary tract, and defecatory disorders [1]. Although pelvic floor disorders are not life-threatening diseases, they significantly negatively impact various aspects of a woman’s life, including social, physical, psychological, occupational, and sexual well-being, ultimately affecting the overall quality of life [2]. Self-administered questionnaires have been suggested as tools for assessing the presence, severity, and impact of symptoms caused by pelvic floor disorders using a standardized and reproducible method. These instruments help to evaluate their effects on a patient’s quality of life and daily activities [3]. Measuring the severity and impact of pelvic floor disorders on affected women is essential, and utilizing questionnaires to assess pelvic floor disorder (PFD) symptoms plays a crucial role in this evaluation [4]. We selected the Pelvic Floor Distress Inventory-20 (PFDI-20) and the Pelvic Floor Impact Questionnaire-7 (PFIQ-7), both of which have been extensively validated in their original language and both the full and short versions have been subsequently translated into multiple languages around the world [5]. The PFDI-20 consists of 20 items divided into three subscales, each assessing symptoms and the level of bother related to different aspects of pelvic floor dysfunction: pelvic organ prolapse symptoms (Pelvic Organ Prolapse Distress Inventory-6, POPDI-6), colorectal symptoms (Colorectal–Anal Distress Inventory-8, CRADI-8), and urinary/bladder symptoms (Urinary Distress Inventory-6, UDI-6) [6]. The PFIQ-7 consists of 21 items distributed across three corresponding subscales—Urinary Impact Questionnaire-7 (UIQ-7), Colorectal–Anal Impact Questionnaire-7 (CRAIQ-7), and Pelvic Organ Prolapse Impact Questionnaire-7 (POPIQ-7). These subscales assess health-related quality of life (HRQoL) concerning the symptoms and associated distress identified in the PFDI-20 [7]. Both questionnaires have been proven to be psychometrically sound, demonstrating validity and reliability [8]. The PFDI and PFIQ, when used together, enable clinicians and researchers to assess the impact of the lower urinary tract, lower gastrointestinal tract, and pelvic organ prolapse symptoms on the quality of life of women with pelvic floor disorders [9]. In Hungary, the availability of validated patient-reported outcome measures in the field of urogynecology remains limited. Only a few instruments have been adapted and validated for Hungarian-speaking populations, including the International Consultation on Incontinence Questionnaire (ICIQ) - Urinary Incontinence Short Form Questionnaire [10], the Australian Pelvic Floor Questionnaire [11], the PISQ-IR (Pelvic Organ Prolapse/Incontinence Sexual Questionnaire – International Urogynecological Assosiation (IUGA) Revised) [12], and the Hungarian version of the Female Sexual Function Index (FSFI-H) [13]. This scarcity underscores the need for further localization and validation of internationally recognized assessment tools to support both clinical decision-making and research in pelvic floor disorders. The aim of the study was to evaluate the reliability, validity, and interpretability of the two questionnaires into the Hungarian language among women experiencing symptoms of pelvic floor dysfunction.

## Materials and Methods

### Study Design and Setting

This study concerns the validation of assessment instruments and was conducted at the Urogynecology Outpatient Clinic of the Department of Obstetrics and Gynecology, University of Debrecen. The validation study was carried out between February 1, 2022, and December 31, 2023.

### Participants

Female patients with confirmed pelvic floor disorders—including urinary incontinence and/or pelvic organ prolapse—were recruited for the study. Inclusion criteria included the presence of at least one diagnosed pelvic floor dysfunction with symptoms persisting for a minimum of 3 months. Exclusion criteria were pregnancy, postpartum status of less than 6 months, a history of pelvic floor surgery within the past 2 years, active malignant disease, intellectual disability, or clinically diagnosed dementia.

### PFDI-20 and PFIQ-7 Questionnaires

The PFDI-20 is composed of three subscales: the Pelvic Organ Prolapse Distress Inventory (POPDI-6), the Colorectal-Anal Distress Inventory (CRADI-8), and the Urinary Distress Inventory (UDI-6). Participants rate the degree of distress caused by each symptom using a scale ranging from 0 to 4, where “0” represents “no” (i.e., the symptom is absent), “1” signifies “not at all” (symptom is present but not bothersome), and “4” corresponds to “quite a bit” (symptom is present and causes significant bother) [14]. The PFIQ-7 evaluates the extent to which bladder, bowel, and vaginal symptoms interfere with various aspects of daily life, including physical activities, travel, social interaction, and emotional well-being. It consists of three subscales: the Urinary Impact Questionnaire (UIQ-7), the Colorectal-Anal Impact Questionnaire (CRAIQ-7), and the Pelvic Organ Prolapse Impact Questionnaire (POPIQ-7). Items are scored on a four-point scale from 0 (“not at all”) to 3 (“quite a bit”), indicating the perceived impact of symptoms on quality of life [15].

### Translation

The translation of the original English-language questionnaire into Hungarian was conducted in accordance with established international guidelines for cross-cultural adaptation of self-report measures. The process followed a rigorous, multi-phase approach to ensure semantic, conceptual, and cultural equivalence between the source and target language versions. In the first phase, two independent forward translations were performed by native Hungarian speakers who were also fluent in English and experienced in medical or psychological terminology. Each translator produced an individual version of the questionnaire without consulting one another. These two initial translations were then compared and reconciled during a structured consensus meeting involving both translators and members of the research team. The aim of this step was to synthesize a single, harmonized Hungarian version that best captured the intended meaning of each item while maintaining clarity and cultural appropriateness. The reconciled Hungarian version was then subjected to a backward translation into English by a native English speaker proficient in Hungarian, who was blinded to the original questionnaire and had no prior involvement in the project. This back-translation was critically reviewed in a second consensus meeting, which included the back-translator, members of the clinical research team, and bilingual experts. The purpose of this comparison was to evaluate the conceptual equivalence between the back-translated and original English versions, identify any inconsistencies or semantic deviations, and refine the Hungarian version accordingly. Any discrepancies or ambiguities identified during the review process were discussed in detail, and appropriate modifications were made to enhance the linguistic accuracy, readability, and cultural relevance of the translated items. This iterative process resulted in a finalized pretest version of the Hungarian questionnaire suitable for subsequent cognitive debriefing and psychometric validation [16].

### Instruments

A total of 150 female patients with clinically confirmed symptoms of pelvic floor disorders, such as urinary incontinence or pelvic organ prolapse, were invited to complete the Hungarian versions of the Pelvic Floor Distress Inventory – Short Form 20 (PFDI-20) and the Pelvic Floor Impact Questionnaire – Short Form 7 (PFIQ-7). The PFDI-20 consisted of 20 items, whereas the PFIQ-7 contained 21 items. Accordingly, a minimum sample size of 105 participants (21 x 5) was required to meet the recommended subject-to-item ratio. Our final sample size of 150 therefore exceeds this threshold and is consistent with established methodological standards [17]. The presence and severity of prolapse-related symptoms were assessed using the Pelvic Organ Prolapse Quantification (POP-Q) system. Before completing the questionnaires, participants received oral and written information about the study and provided written informed consent. To evaluate test–retest reliability, a subgroup of 50 patients completed both questionnaires again after a 2-week interval. During this period, no therapeutic intervention was administered to maintain symptom stability. To assess construct validity, participants also completed the SF-36 Health Survey. The results were used to examine the correlation between the SF-36 scores and those of the Hungarian versions of the PFDI-20 and PFIQ-7. The 36-item Short Form Health Survey (SF-36) is a validated, generic measure designed to evaluate health-related quality of life across diverse populations and disease conditions. It encompasses 36 items aggregated into eight domains: physical functioning, role limitations due to physical health problems, bodily pain, general health perceptions, vitality (energy/fatigue), social functioning, role limitations due to emotional problems, and mental health. Each domain is scored on a scale from 0 to 100, with higher scores reflecting more favorable health status [18]. The SF-36 enables cross-population comparisons and is commonly employed in construct validation of condition-specific instruments. The SF-36 has previously been adapted for use in the Hungarian population.

### Statistical Analysis

The Hungarian versions of the PFDI-20 and PFIQ-7 were evaluated for their validity and reliability. To evaluate the psychometric properties of the questionnaires, internal consistency was measured using Cronbach’s alpha, while test–retest reliability was assessed by calculating intraclass correlation coefficients (ICC). Construct validity was further examined using Spearman’s rank correlation coefficient, a nonparametric test that assesses the strength and direction of monotonic associations between variables. This method is particularly suitable when the data are not normally distributed or are ordinal. Spearman correlations were calculated between the Hungarian versions of the PFDI-20 and PFIQ-7 scores and the SF-36 Health Survey domains to evaluate convergent validity.

## Results

Between February 1, 2022 and December 31, 2023, 150 patients participated in the study by completing the Hungarian versions of the PFDI-20 and PFIQ-7 questionnaire and the SF-36 Health survey for psychometric evaluation.

### Demographic and Clinical Characteristics

Demographic characteristics collected for each participant included age, gravidity, parity, the number of spontaneous vaginal deliveries, and body mass index (BMI) calculated from self-reported height and weight. BMI was calculate using the standard formula: weight in kilograms divided by height in meters squared (kg/m^2^) (Table 1).

**Table 1:** Demographic and clinical characteristics of the participants.

	Average	SD
Age	59.07	±11.13
Gravidity	2.97	±1.56
Parity	2.17	±0.92
Spontaneous vaginal delivery	1.83	±1.13
BMI	27.51	±4.62

### Internal Consistency

The Hungarian versions of the PFDI-20 and PFIQ-7 demonstrated high levels of internal consistency. The Cronbach’s alpha coefficient for the PFDI-20 was 0.885, with confidence interval from 0.8576 to 0.9048 (Table 2). For the PFIQ-7, the overall alpha was 0.939, with confidence interval from 0.9188 to 0.9528 (Table 3). These values indicate a strong interrelatedness among items within each scale.

**Table 2:** Cronbach’s alpha values indicate the internal consistency of the PFDI-20 subscales

	Cronbach’s alpha	Confidence interval
POPDI	0.7126	0.6370–0.7706
CRADI	0.8565	0.8187–0.8835
UDI	0.8546	0.8133–0.8872
PFDI	0.8851	0.8576–0.9048

**Table 3:** Internal consistency of the PFIQ-7 and its subscales, as assessed by Cronbach’s alpha

	Cronbach’s alpha	Confidence interval
UIQ	0.9255	0.9037–0.9441
POPIQ	0.9369	0.9067–0.9577
CRAIQ	0.9374	0.9189–0.9542
PFIQ	0.9387	0.9188–0.9528

### Test–Retest Reliability

Test–retest reliability, as assessed by intraclass correlation coefficients (ICC), was also high, 0.903 (*p* < 0.01) for the PFDI-20 (Table 4) and 0.933 (*p* < 0.01) for the PFIQ-7 (Table 5), reflecting good temporal stability of the instruments.

**Table 4:** The table presents the test–retest reliability of the PFDI-20 questionnaire based on intraclass correlation coefficients (ICCs)

	POPDI	CRADI	UDI	PFDI-20
ICC	0.903	0.866	0.896	0.926

**Table 5:** This table illustrates the test–retest reliability of the PFIQ-7 questionnaire, measured using intraclass correlation coefficients (ICCs)

	UIQ	POPIQ	CRAIQ	PFIQ
ICC	0.933	0.965	0.9	0.949

### Spearman’s Rank Correlation

The Spearman’s rank correlation between the SF-36 Health Survey and the Hungarian version of the PFDI-20 was *r* = −0.4379 (*p* < 0.01), while with the Hungarian version of the PFIQ-7 it was *r* = −0.5618 (*p* < 0.01). The strongest correlations were observed with the UDI dimensions: in the PFDI-20, the UDI showed a correlation of *r* = −0.4342 (*p* < 0.01), and in the PFIQ-7, the UIQ dimension had a correlation of *r* = −0.5368 (Table 6).

**Table 6:** Spearman correlation analyses were conducted to examine the relationships between scores on the PFDI-20, PFIQ-7, and SF-36 instruments

	POPDI-6 subscales score	CRADI-8 subscales score	UDI-6 subscales score	PFDI-20 total score	UIQ-7 subscales score	POPIQ-7 subscales score	CRAIQ-7 subscales score	PFIQ-7 total score	SF-36 total score
POPDI-6 subscales score	1	0.3642	0.5047	0.7949	0.4558	0.3528	0.5588	0.5925	−0.2710
CRADI-8 subscales score	–	1	0.3648	0.6646	0.3499	0.5565	0.2753	0.4723	−0.2837
UDI-6 subscales score	–	–	1	0.8439	0.7431	0.3298	0.2726	0.6286	−0.4342
PFDI-20 total score	–	–	–	1	0.6858	0.5232	0.4658	0.7380	−0.4379
UIQ-7 subscales score	–	–	–	–	1	0.4096	0.3288	0.8149	−0.5368
POPIQ-7 subscales score	–	–	–	–	–	1	0.4048	0.6394	−0.3683
CRAIQ-7 subscales score	–	–	–	–	–	–	1	0.7506	−0.3445
PFIQ-7 total score	–	–	–	–	–	–	–	1	−0.5618
SF-36 total score	–	–	–	–	–	–	–	–	1

### POP-Q Stages

All participants underwent POP-Q assessment. The distribution of prolapse severity according to POP-Q staging was as follows: 21.77% of women were classified as stage I, 31.45% as stage II, 44.84% as stage III, and 4.84% as stage IV. This distribution reflects a study population covering the full spectrum of prolapse severity, with the largest proportion of women presenting with stage III prolapse. POPQ stage and POPDI scores showed a nonsignificant weak positive correlation (*r* = 0.12, *p* = 0.21).

## Discussion

In our current study, our research group successfully translated and validated PFDI-20 and PFIQ-7 into the Hungarian language, which would be available for daily use for Hungarian Urogynecologist specialist for research and consultation purposes. This article discusses female pelvic floor disorders (PFDs), which include urinary and fecal incontinence, pelvic organ prolapse, and other dysfunctions that significantly impact women’s quality of life. Self-administered questionnaires, such as the Pelvic Floor Distress Inventory-20 (PFDI-20) and the Pelvic Floor Impact Questionnaire-7 (PFIQ-7), are widely used to assess the severity and impact of these conditions. These validated tools measure symptoms and health-related quality of life (HRQoL) across different pelvic floor dysfunction aspects [19]. The study evaluates these questionnaires’ reliability, validity, and interpretability among Hungarian women, who are dominantly Caucasian with catholic religious background, demonstrating PFD symptoms. The results also confirmed the construct validity of the Hungarian versions through correlations with a validated general health-related quality of life instrument, assessed by the SF-36 Health Survey. Our results demonstrating negative correlations between severe pelvic floor symptoms and lower perceived quality of life align with our expectations, and the currently available data in the literature [20]. Among the subscales, urinary symptoms appeared to have the greatest influence on quality-of-life measures, as reflected by the strongest correlations with relevant SF-36 domains. This finding is consistent with the clinical understanding that urinary symptoms, particularly incontinence, often lead to significant psychosocial and functional limitations for affected women. The translation and cultural adaptation of the instruments followed a rigorous, multi-step process that included independent forward and backward translations, consensus reviews, and semantic validation [21]. This ensured that the Hungarian versions preserved the conceptual and linguistic integrity of the original instruments while maintaining cultural relevance. The study was conducted in a clinical setting with well-defined inclusion and exclusion criteria, and participants completed both condition-specific and general quality-of-life questionnaires. A subset of participants also completed the instruments again after a defined interval without undergoing treatment during this period, allowing for a robust assessment of temporal reliability. In summary, the findings indicate that the Hungarian versions of the PFDI-20 and PFIQ-7 are reliable and valid tools for assessing symptom burden and quality of life in women with pelvic floor disorders. Their use in both clinical practice and research may contribute to better symptom assessment and improved patient care in the Hungarian-speaking population. One of the strengths of our study was the relatively large sample size. This allowed for greater statistical power and increased the reliability of our findings. A limitation of the study is that the data originated from a single institution, although, as a tertiary care center, it served a population spanning multiple European regions. The data that support the findings of this study are available from the corresponding author upon request.

## Data Availability

Statement is added to the text.

## References

[1] Weber AM, Abrams P, Brubaker L, Cundiff G, Davis G, Dmochowski RR, et al. The standardization of terminology for researchers in female pelvic floor disorders. Int Urogynecol J. 2001;12(3):178–86. 10.1007/PL00004033 PMC2815805 11451006

[2] Sánchez-Sánchez B, Torres-Lacomba M, Yuste-Sánchez MJ, Navarro-Brazález B, Pacheco-da-Costa S, Gutiérrez-Ortega C, et al. Cultural adaptation and validation of the Pelvic Floor Distress Inventory short form (PFDI-20) and Pelvic Floor Impact Questionnaire short form (PFIQ-7) Spanish versions. Eur J Obstet Gynecol Reproduct Biol. 2013;170(1):281–5. 10.1016/j.ejogrb.2013.05.009 10.1016/j.ejogrb.2013.07.006 23891390

[3] Naughton MJ, Donovan J, Badia X, Corcos J, Gotoh M, Kelleher C, et al. Symptom severity and QOL scales for urinary incontinence. Gastroenterology. 2004;126(1 Suppl 1):S114. 14978647 10.1053/j.gastro.2003.10.059

[4] Arouca MAF, Duarte TB, Lott DAM, Magnani PS, Nogueira AA, Rosa-E-Silva JC, et al. Validation and cultural translation for Brazilian Portuguese version of the Pelvic Floor Impact Questionnaire (PFIQ-7) and Pelvic Floor Distress Inventory (PFDI-20). Int Urogynecol J. 2016;27(7):1097–106. 10.1007/s00192-015-2930-z 26782099 10.1007/s00192-015-2938-8

[5] Barber MD, Kuchibhatla MN, Pieper CF, Bump RC. Psychometric evaluation of two comprehensive condition-specific quality of life instruments for women with pelvic floor disorders. Am J Obstet Gynecol. 2001;185(6):1388–95. 10.1067/mob.2001.119078 11744914 10.1067/mob.2001.118659

[6] Teig CJ, Grotle M, Bond MJ, Prinsen CAC, Engh MAE, Cvancarova MS. Norwegian translation, and validation, of the Pelvic Floor Distress Inventory (PFDI-20) and the Pelvic Floor Impact Questionnaire (PFIQ-7). Int Urogynecol J. 2017;28(7):1005–17. 10.1007/s00192-016-3209-z. 28062903 10.1007/s00192-016-3209-z

[7] Due U, Brostrøm S, Lose G. Validation of the Pelvic Floor Distress Inventory-20 and the Pelvic Floor Impact Questionnaire-7 in Danish women with pelvic organ prolapse. Acta Obstet Gynecol Scand. 2013;92(9):1041–8. 10.1111/aogs.12189. 23725572 10.1111/aogs.12189

[8] Kaplan PB, Sut N, Sut HK. Validation, cultural adaptation and responsiveness of two pelvic-floor-specific quality-of-life questionnaires, PFDI-20 and PFIQ-7, in a Turkish population. Eur J Obstet Gynecol Reprod Biol. 2012;162(2):229–33. 10.1016/j.ejogrb.2012.03.004. 22480412 10.1016/j.ejogrb.2012.03.004

[9] Barber MD, Walters MD, Bump RC. Short forms of two condition-specific quality-of-life questionnaires for women with pelvic floor disorders (PFDI-20 and PFIQ-7). Am J Obstet Gynecol. 2005;193(1):103–13. 10.1016/j.ajog.2004.12.025. 16021067 10.1016/j.ajog.2004.12.025

[10] Kenyeres B, Sarlós DP, Szanto Á, Pytel Á. Validation of the Hungarian version of the International Consultation on Incontinence Questionnaire – Urinary Incontinence Short Form (ICIQ-UI/SF) in females with lower urinary tract symptoms. Eur Urol Suppl. 2015;14:e1256. 10.1016/S1569-9056(15)30293-1.

[11] Hock M, Tiringer I, Ambrus E, Németh Z, Farkas B. Validation and translation of the Hungarian version of the Australian Pelvic Floor Questionnaire (APFQ-H). Int Urogynecol J. 2023;34(6):1187–94. 10.1007/s00192-022-05322-2. 35972523 10.1007/s00192-022-05322-2 PMC10238311

[12] Farkas B, Tiringer I, Farkas N, Kenyeres B, Németh Z. Hungarian language validation of the Pelvic Organ Prolapse/Incontinence Sexual Questionnaire, IUGA-Revised (PISQ-IR). Int Urogynecol J. 2016;27(12):1831–6. 10.1007/s00192-016-3047-z. 27230407 10.1007/s00192-016-3047-z

[13] Hock M, Farkas N, Tiringer I, Gitta S, Németh Z, Farkas B. Validation and translation of the Hungarian version of the Female Sexual Function Index (FSFI-H). Int Urogynecol J. 2019;30(12):2109–20. 10.1007/s00192-019-04049-x. 31359116 10.1007/s00192-019-04049-x PMC6861199

[14] de Arruda GT, de Andrade DF, Virtuoso JF. Internal structure and classification of pelvic floor dysfunction distress by PFDI-20 total score. J Patient-Report Outcomes. 2022;6(1):51. 10.1186/s41687-022-00459-6 10.1186/s41687-022-00459-6 PMC9109438 35576026

[15] Molina-Barea R, Slim M, Calandre EP. Health-related quality of life and psychosocial variables in women with colorectal pelvic floor dysfunction: a cross-sectional study. Healthcare. 2024;12(6):668. 10.3390/healthcare12060668. 38540632 10.3390/healthcare12060668 PMC10970336

[16] Guillemin F, Bombardier C, Beaton D. Cross-cultural adaptation of health-related quality of life measures: literature review and proposed guidelines. J Clin Epidemiol. 1993;46(12):1417–32. 10.1016/0895-4356(93)90142-N. 8263569 10.1016/0895-4356(93)90142-n

[17] Chan SS, Cheung RY, Yiu AK, Li JC, Lai BP, Choy KW, et al. Chinese validation of Pelvic Floor Distress Inventory and Pelvic Floor Impact Questionnaire. Int Urogynecol J. 2011;22(10):1305–12. 10.1007/s00192-011-1450-z 21611791 10.1007/s00192-011-1450-z

[18] Brazier JE, Harper R, Jones NM, O’Cathain A, Thomas KJ, Usherwood T, et al. Validating the SF-36 health survey questionnaire: new outcome measure for primary care. BMJ. 1992;305(6846):160–4. 10.1136/bmj.305.6846.160 1285753 10.1136/bmj.305.6846.160 PMC1883187

[19] Jensen JE, Ngobi MD, Kiweewa FM, Fleecs JD, Vemulapalli R, Steffen HA, et al. Reliability and validation of the PFIQ-7 and PFDI-20 in the Luganda language. Int Urogynecol J. 2024;35(8):1681–7. 10.1007/s00192-024-05866-5 38995423 10.1007/s00192-024-05866-5 PMC11380631

[20] Chen W, Gong J, Liu M, Cai YC. Long-term health outcomes and quality of life in women with untreated pelvic floor dysfunction: a single-center cohort study. Front Public Health. 2025;12:1495679. 10.3389/fpubh.2024.1495679. 39839434 10.3389/fpubh.2024.1495679 PMC11746105

[21] Melkie TB, Gashaw ZM, Workineh ZA, Andargie TM, Debele TZ, Nigatu SG. Translation, reliability, and validity of Amharic versions of the Pelvic Floor Distress Inventory (PFDI-20) and Pelvic Floor Impact Questionnaire (PFIQ-7). PLoS One. 2022;17(11):e0270434. 10.1371/journal.pone.0270434. 36395162 10.1371/journal.pone.0270434 PMC9671332

